# Biomechanical analysis of single-level interbody fusion with different internal fixation rod materials: a finite element analysis

**DOI:** 10.1186/s12891-020-3111-1

**Published:** 2020-02-14

**Authors:** Yueh-Ying Hsieh, Fon-Yih Tsuang, Yi-Jie Kuo, Chia-Hsien Chen, Chang-Jung Chiang, Chun-Li Lin

**Affiliations:** 1 Department of Biomedical Engineering, National Yang-Ming University, Taipei, Taiwan; 2 Department of Orthopedics, Shuang Ho Hospital, Taipei Medical University, New Taipei City, Taiwan; 3 Department of Orthopedic Surgery, School of Medicine, College of Medicine, Taipei Medical University, Taipei, Taiwan; 4 Division of Neurosurgery, Department of Surgery, National Taiwan University Hospital, Taipei, Taiwan; 5 Department of Traumatology, National Taiwan University Hospital, Taipei, Taiwan; 6 Institute of Biomedical Engineering, National Taiwan University, Taipei, Taiwan; 7 Department of Orthopedic Surgery, Taipei Municipal Wanfang Hospital, Taipei Medical University, Taipei, Taiwan; 8 Graduate Institute of Biomedical Materials and Tissue Engineering, College of Biomedical Engineering, Taipei Medical University, Taipei, Taiwan

**Keywords:** Spinal interbody fusion, Flexible rods, Finite element analysis, Biomechanical study

## Abstract

**Background:**

Lumbar spinal fusion with rigid spinal fixators as one of the high risk factors related to adjacent-segment failure. The purpose of this study is to investigate how the material properties of spinal fixation rods influence the biomechanical behavior at the instrumented and adjacent levels through the use of the finite element method.

**Methods:**

Five finite element models were constructed in our study to simulate the human spine pre- and post-surgery. For the four post-surgical models, the spines were implanted with rods made of three different materials: (i) titanium rod, (ii) PEEK rod with interbody PEEK cage, (iii) Biodegradable rod with interbody PEEK cage, and (iv) PEEK cage without pedicle screw fixation (no rods).

**Results:**

Fusion of the lumbar spine using PEEK or biodegradable rods allowed a similar ROM at both the fusion and adjacent levels under all conditions. The models with PEEK and biodegradable rods also showed a similar increase in contact forces at adjacent facet joints, but both were less than the model with a titanium rod.

**Conclusions:**

Flexible rods or cages with non-instrumented fusion can mitigate the increased contact forces on adjacent facet joints typically found following spinal fixation, and could also reduce the level of stress shielding at the bone graft.

## Introduction

Posterior instrumentation with pedicle screw fixations has been shown to provide immediate rigid fixation and increase the rate of spinal fusion [1, 2]. However, the high rigidity of pedicle screw systems may lead to adjacent segment diseases (ASDs) and hardware-related discomfort. Several clinical studies have implicated lumbar spinal fusion with rigid spinal fixators as one of the high risk factors related to adjacent-segment failure [3–6].

On the other hand, biomechanical studies have shown that fusion at one or two levels can increase the stress at adjacent segments [7–9]. Studies [9, 10] have also indicated that the fusion surgery might increase the stress at facet joints and increase segmental mobility and intradiscal pressure at adjacent levels. Such abnormal loading on the spine may accelerate degeneration of the facet joints. Most notably, these factors can be mitigated or corrected to some degree during surgery, and thus have the potential to improve the patient outcome.

In theory, increased mechanical stress at adjacent segments may accelerate their degeneration. Flexible polymer rods were developed to reduce abnormal mechanical stress, hardware-related discomfort, and some metal hypersensitivity [11, 12]. De Lure et al. [12] reviewed 30 cases of interbody fusion with PEEK rods as posterior spinal fixators. After an average of 18 months follow-up, there was no evidence of adjacent segment diseases in any of the cases. In contrast to traditional metallic implants, some polymer materials have biodegradable properties that allow the implant to degrade gradually over time [13, 14]. The Young’s modulus of the polymer rods was found to be closer to that of bone, and the lower stiffness of the rods meant less gradual dynamic loading and stress shielding of the fusion site.

Due to the association between rigid spinal fixators and ASDs, some surgeons have pointed out that patients may not need rigid instrumentation permanently implanted after spinal fusion had occurred. In order to mitigate ASDs, the spinal implants may be removed as early as possible once the fusion process has finished. Hsieh et al. [15] used a lumbosacral model to evaluate disc stresses, facet loads and range of motion (ROM) of the adjacent segments after posterior instrumentation. Their study concluded that the removal of spinal fixation after complete spinal fusion might mitigate the pathological changes at adjacent segments. Jeon et al. [16] used radiological and clinical data to evaluate the benefits of removing pedicle screws after fusion, finding that removing the spinal fixation could significantly alleviate patients’ disability and pain.

Although using semi-rigid spinal fixators appears to decrease the occurrence of adjacent segment diseases, there are still a number of uncertainties concerning the biomechanical behavior of the implanted lumbar spine. The purpose of our study is to investigate the biomechanical behavior of the lumbar spine after the interbody fusion process has finished. The lumbar spines were implanted with three different rod materials: biodegradable rods, PEEK rods, and titanium rods. The effect on adjacent segments under different physiological loading conditions was also simulated.

## Materials and methods

**Fig. 1:**
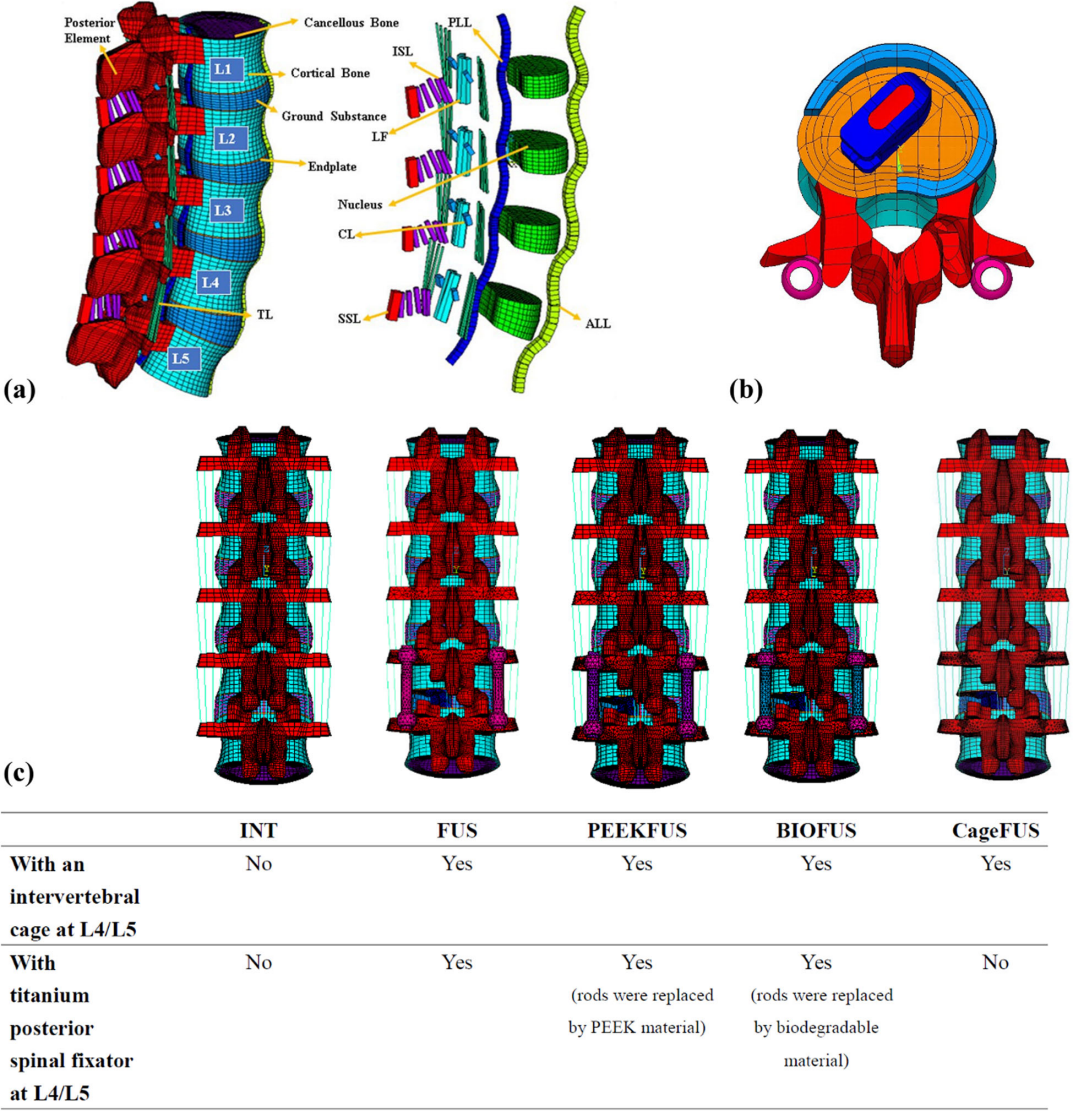
FE models of the spine with and without implants. **a** The osseous structures, intervertebral discs, and ligaments of the intact spine. **b** At the L4-L5 disc space, the cage was placed obliquely with the removal of left posterolateral corner of the annulus fibrosus, as in TLIF procedures. **c** Five FE models used in this study

Previous studies by the authors developed a finite element model of an intact lumbar spine in ANSYS 14.0 (ANSYS Inc., Canonsburg, PA, USA) [17–19], including osseoligamentous L1-L5 vertebrae, endplates, intervertebral discs, posterior bony elements, and all 7 ligaments (Fig. 1a). The intervertebral discs contained a nucleus pulposus and annulus fibrosus, with 12 double-cross-linked fiber layers embedded in the ground substance. The annulus material was modeled based on a hyperelastic, incompressible, 2-parameter (C1, C2) Mooney-Rivlin formulation, and the nucleus pulposus was established as an incompressible fluid. Convergence testing and validation of the intact model were completed in previous studies [18, 19], with the results being similar to other published finite element models [20]. The study of Dreischarf et al. [20] also revealed that our finite element models can be used as an improved predictive tool in order to estimate the response of the lumbar spine using different motion input for various cases analyzed. Details of the intact model and its material properties were described in previous studies [17, 18].

This study simulated a CB PROT II Posterior Spinal Fixation (Chin Bone Tech. Corp, Taiwan; US FDA 510(k): K142655) with titanium alloy, PEEK, and biodegradable rods. The pedicle screws were made of Ti-6Al-4 V. The lumbar intervertebral cage ReBorn Essence (New Taipei City, Baui Biotech, Co., Ltd., Taiwan) made from PEEK was used to simulate interbody fusion. The cage was implanted through the posterolateral approach and crossed the coronal midline. The posterolateral corner at the left side of the L4-L5 annulus fibrosus was also removed to simulate the condition after a transforaminal lumbar interbody fusion procedure [21] (Fig. 1b). Interfaces between the cage and bone grafts were bonded. Three different types of rods, intervertebral cages and pedicle screws were meshed using 8-node solid elements. The disc at the fusion level was replaced by a cage and was bridged with pedicle screws and rods. The interfaces between facet articular surfaces were defined as standard contact pairs at all levels. The lumbar spine model was fixed at the base of the fifth vertebrae. A hybrid method detailed by Panjabi was used to evaluate the effect of single-level interbody fusion on the adjacent segments [22].

Five models (Fig. 1c) were developed in this study: (1) INT: intact spine without any implants, (2) FUS: spine implanted with a lumbar cage and pedicle screws with a Ti-6Al-4 V rod system at L4-L5, (3) PEEKFUS: spine implanted with a lumbar cage and pedicle screws with a PEEK rod system at L4-L5, (4) BIOFUS: spine implanted with a lumbar cage and pedicle screws with a biodegradable rod (Young modulus: 6.6 GPa, Possion ratio: 0.29) system at L4-L5, (5) CageFUS: spine implanted with a lumbar intervertebral cage at L4-L5 without pedicle screws or rods (interbody fusion without pedicle screw system).

**Fig. 2:**
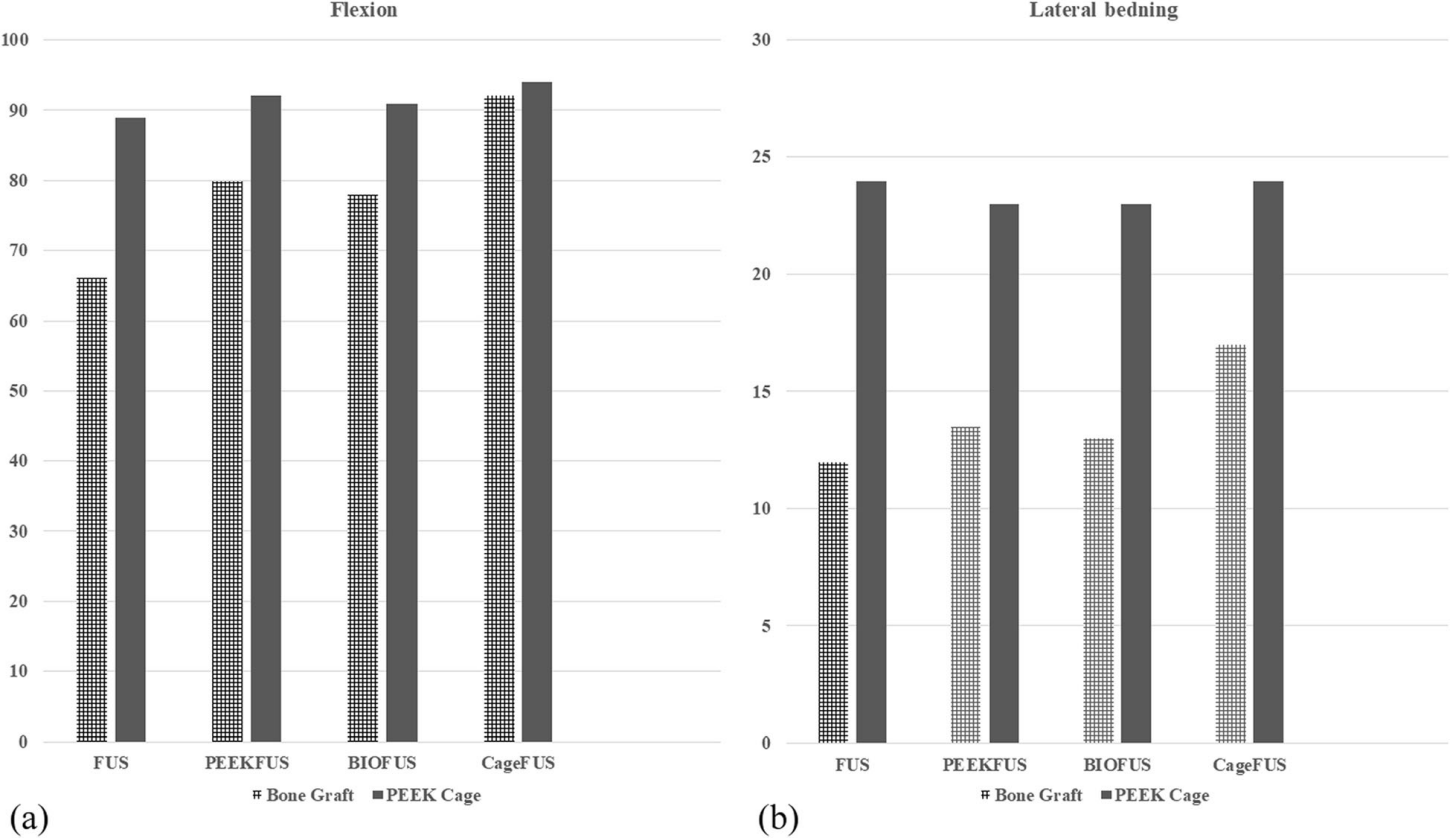
Loading (N) on cage and bone grafts in each group. **a** in flexion and **b** in lateral bending

Loading on the models was applied in two steps. First, an axial load of 150 N was applied perpendicular to the upper endplate of L1, this axial load with the displacement-controlled method was more clinically relevant in evaluating the fusion model at the adjacent levels [23]. Second, a pure unconstrained moment was applied in 0.36 Nm increments to ensure the resultant ROM (L1 to L5) of all finite element models would equal the motion corresponding to 9 degrees in extension, 16 degrees in flexion, 22 degrees in left lateral bending, and 17 degrees in left torsion. The resultant ranges of motion of the instrumented level, the level adjacent to the fusion site, and the whole lumbar spine are listed in Table 1, as well as the resultant moment and segmental stiffness of each model. The peak disc stresses and the facet contact forces at L2–3/L3–4 under extension, flexion, torsion, and left lateral bending for all models were also recorded for comparison. All ranges of motion, contact forces, and intradiscal pressures in the spinal models were normalized with respect to the values attained for the intact spine. Figure 2 shows loading on the lumbar cage and bone graft in each fusion model under different loading conditions.

**Table 1:** ROM of five FE models at all motion segments

Motion	Model	L1-L2 (Degree)	L2-L3 (Degree)	L3-L4 (Degree)	L4-L5 (Degree)	Moment (N.m)	L1-L5 Stiffness (N.m/Degree)
Flexion	INT	4.45 (100%)	4.43 (100%)	4.34 (100%)	5.78 (100%)	8.7 (100%)	0.46 (100%)
	FUS	5.66 (127%)	5.65 (128%)	6.78 (156%)	1.01 (17%)	11.1 (128%)	0.58 (126%)
	PEEKFUS	5.56 (125%)	5.59 (126%)	6.66 (153%)	1.17 (20%)	10.9 (125%)	0.57 (124%)
	BIOFUS	5.56 (125%)	5.54 (125%)	6.61 (152%)	1.26 (22%)	10.8 (124%)	0.57 (124%)
	CageFUS	5.40 (121%)	5.38 (121%)	6.32 (146%)	1.65 (29%)	10.3 (118%)	0.55 (120%)
Extension	INT	3.05 (100%)	2.62 (100%)	2.56 (100%)	2.57 (100%)	7.80 (100%)	0.72 (100%)
	FUS	3.60 (118%)	3.11 (119%)	3.19 (125%)	0.84 (33%)	9.60 (123%)	0.89 (124%)
	PEEKFUS	3.60 (118%)	3.11 (119%)	3.19 (125%)	0.87 (34%)	9.60 (123%)	0.89 (124%)
	BIOFUS	3.59 (118%)	3.11 (119%)	3.20 (125%)	0.87 (34%)	9.60 (123%)	0.89 (124%)
	CageFUS	3.58 (117%)	3.09 (118%)	3.17 (124%)	1.01 (39%)	9.60 (123%)	0.87 (121%)
Lateral bending	INT	5.74 (100%)	5.01 (100%)	4.70 (100%)	4.48 (100%)	9.90 (100%)	0.50 (100%)
	FUS	8.14 (142%)	5.48 (109%)	5.11 (109%)	0.85 (19%)	9.90 (100%)	0.51 (102%)
	PEEKFUS	7.97 (139%)	5.36 (107%)	5.02 (107%)	1.15 (26%)	9.66 (98%)	0.50 (99%)
	BIOFUS	7.91 (138%)	5.32 (106%)	4.95 (105%)	1.28 (29%)	9.6 (97%)	0.49 (98%)
	CageFUS	7.86 (137%)	5.23 (104%)	4.85 (103%)	1.82 (41%)	9.58 (97%)	0.49 (98%)
Torsion	INT	2.01 (100%)	2.30 (100%)	2.68 (100%)	3.75 (100%)	9.90 (100%)	0.92 (100%)
	FUS	4.84 (241%)	2.23 (97%)	2.54 (95%)	1.14 (30%)	8.70 (88%)	0.81 (88%)
	PEEKFUS	4.38 (218%)	2.07 (90%)	2.39 (89%)	1.86 (50%)	7.80 (79%)	0.73 (79%)
	BIOFUS	4.38 (218%)	2.07 (90%)	2.39 (89%)	1.86 (50%)	7.80 (79%)	0.73 (79%)
	CageFUS	4.18 (208%)	1.96 (85%)	2.33 (87%)	2.55 (68%)	7.42 (75%)	0.67 (73%)

The percentages indicate the ROM of all models normalized by the ROM of the INT model

## Results

### Range of motion of each level

Table 1 shows that the ROM increased at adjacent segments and decreased at the fusion level in all but the intact (INT) model. When the data from all implanted models was normalized to the mean of the control group (intact model), each instrumented model had a similar ROM under the various loading conditions.

### Contact force at adjacent facet joints

Table 2 shows how the contact force ratios at the adjacent facet joints at L2–3 and L3–4 levels increased under the various loading conditions. The increase in contact force ratios at the L3–4 facets was greater than that at the L2–3 facets in all fusion models. The greatest increase occurred in the FUS model, and was followed closely by the PEEKFUS and BIOFUS models. The smallest change in contact force occurred in the CageFUS model, where the contact forces on adjacent facet joints were similar to the intact model.

**Table 2:** Facet joint forces at instrumented levels and cephalic adjacent levels

Motion	Model	L2-L3 (N)	L3-L4 (N)	L4-L5
Extension	INT	65 (100%)	71 (100%)	66 (100%)
	FUS	82 (126%)	90 (127%)	0 (0%)
	PEEKFUS	82 (126%)	90 (127%)	2 (3%)
	BIOFUS	82 (126%)	90 (127%)	3 (5%)
	CageFUS	82 (126%)	90 (127%)	15 (23%)
Lateral bending	INT	19 (100%)	9 (100%)	13 (100%)
	FUS	23 (121%)	21 (233%)	0 (0%)
	PEEKFUS	21 (111%)	18 (200%)	0 (0%)
	BIOFUS	21 (111%)	18 (200%)	0 (0%)
	CageFUS	19.8 (104%)	15 (167%)	7.5 (58%)
Torsion	INT	125 (100%)	124 (100%)	112 (100%)
	FUS	116 (93%)	119 (96%)	1 (1%)
	PEEKFUS	104 (83%)	103 (83%)	45 (40%)
	BIOFUS	104 (83%)	103 (83%)	45 (40%)
	CageFUS	101 (81%)	100 (81%)	106 (95%)

The percentages indicate the facet joint forces of all models normalized by the facet joint forces of the INT model

### Peak stress on intervertebral discs (IVDs)

Table 3 demonstrates the increase in the ratio of peak stress on the IVDs at L2–3 and L3–4 levels under different loading conditions. The peak stress on the adjacent disc was significantly higher in all fusion models than in the INT model. The L3–4 level also showed a greater increase than the L2–3 level for all fusion models. The FUS model demonstrated that the greatest change in stress at the IVDs (at both L2–3 and L3–4 levels), while the CageFUS model had the smallest increase. Using a semi-rigid rod for stabilization (PEEKFUS and BIOFUS) resulted in a lower peak stress on the adjacent disc than with the use of a rigid titanium rod (FUS model), especially under torsion.

**Table 3:** Disc stresses at cephalic adjacent levels

Motion	Model	L2-L3 (kPa)	L3-L4 (kPa)
Flexion	INT	880 (100%)	742 (100%)
	FUS	1100 (125%)	1150 (155%)
	PEEKFUS	1080 (123%)	1140 (154%)
	BIOFUS	1070 (122%)	1120 (151%)
	CageFUS	1070 (122%)	1110 (150%)
Extension	INT	398 (100%)	424 (100%)
	FUS	460 (116%)	525 (124%)
	PEEKFUS	460 (116%)	525 (124%)
	BIOFUS	460 (116%)	524 (124%)
	CageFUS	460 (116%)	524 (124%)
Lateral bending	INT	951 (100%)	906 (100%)
	FUS	1030 (108%)	975 (108%)
	PEEKFUS	1000 (105%)	955 (105%)
	BIOFUS	1000 (105%)	950 (105%)
	CageFUS	995 (105%)	941 (104%)
Torsion	INT	314 (100%)	345 (100%)
	FUS	316 (101%)	355 (103%)
	PEEKFUS	294 (93%)	336 (97%)
	BIOFUS	293 (93%)	335 (97%)
	CageFUS	286 (91%)	327 (95%)

The percentages indicate the disc stresses of all models normalized by the disc stresses of the INT model

### Loading on lumbar cage and bone grafts

Figure 2 shows the forces on the PEEK cages and bone grafts under various loading conditions. The loading on the bone grafts in the PEEKFUS and BIOFUS models exceeded the FUS model immediately after surgery. Under all motions, the loading on the bone grafts in the CageFUS model was greater than in the other fusion models.

## Discussion

Dynamic stabilization systems have been suggested to maintain a certain degree of motion and reduce the occurrence of adjacent segment diseases in comparison to rigid fixation [24–26]. Previous studies have shown that using a semi-rigid fixator reduces the stress at adjacent levels and at the fusion site [27, 28]. Chen et al. [29] also found that sufficient anterior support could lower the risk of failure of the spinal fixation and decrease the requirement for a fully stable posterior pedicle screw system. However, there is limited information on the biomechanical behavior of different rod materials when used for interbody fusion. This study used finite element methods to simulate complete interbody fusion and analyze the biomechanical properties of the fusion site and adjacent levels when implanted with different rod materials. Similar non-fusion models of low stiffness or dynamic devices have been reported in literature [24–26, 28], but few investigations have considered the situation after the fusion process has finished.

Theoretically, constructs with lower rigidity should maintain a certain degree of motion and reduce the stress on facet joints and discs at adjacent levels. In our study, there was no significant difference in the ROM in all fusion models. This shows that the PEEK cage may play an important role in providing initial stability to the fusion site, and at the same time altering the biomechanical behavior at the fusion level and adjacent levels. Spinal cages are known to be more effective than posterior fixation at controlling the biomechanical environment and spinal stability. Ponnappan et al. [30] used a cadaveric model implanted with PEEK and titanium rods to analyze the stability at the fusion level, and reported no significant differences between the two materials for performing interbody fusion with cages. In the CageFUS model, the reduction in ROM at the fusion level was greater in flexion and extension than in bending or rotational motions. This is because the instantaneous center of flexion/extension at the L4–5 level was replaced by the cage, and the local buffer space at the L4–5 level was limited in flexion/extension motions. These results implied that the cage might be the major stabilizer at the fusion level in extension and flexion, and the use of a pedicle screw system increases the stability under bending and rotational motions. The models with less rigid rods (PEEKFUS and BIOFUS) could preserve a greater ROM in rotation and lateral bending when compared with the titanium rod model (FUS). In axial rotation, the results showed reduced stiffness immediately after placing the cage (Cage FUS model). This might be caused by the removal of a section of the annulus during the procedure, which may decrease the stability at the index level despite the presence of a cage. Similar results were reported by Krijnen et al. in their in vitro evaluation using a goat model [31]. Regarding the stiffness of the spine, there was no significant difference among all fusion models. In other words, the use of a PEEK cage may be the primary factor influencing the stiffness of the lumbar spine in single-level interbody fusion, and the rigidity of the rods has less of an impact on the fusion procedure.

There was no significant difference in the results for maximal stress at the adjacent discs was not significantly different among all fusion models, where the relative increase in stress was between 4 and 55% at the L3–4 level and between 5 and 25% at the L2–3 level under all loading conditions except rotation. The stress was much higher in extension and flexion than in bending. Under rotational motions, the stresses at the adjacent level were slightly less than the intact model, which might be caused by the decrease in rotational stability at the fusion site. The greatest increase in disc stress occurred at the L3–4 level in all fusion models under flexion, increasing by up to 50% in the all models. The stress at the L2–3 disc also increased after interbody fusion with a PEEK cage. These results correspond with those of Chen et al. [32], in which finite element methods were used to analyze changes in stress at adjacent discs after the fusion procedure without pedicle screw instrumentation.

The increased stiffness of the index level would typically increase the stress on the fact joints and adjacent disc. However, according to the results of this study, there was no significant change in the stiffness of all fusion models under lateral bending, extension and flexion. Compared with the interbody fusion model without instrumentation, supplementing the support with pedicle screws increased both the peak stress on the adjacent disc and stiffness of the spine.

When the models were placed in flexion, the facet joints separated and lost contact, and therefore no facet contact forces were recorded for flexion. Using rods with lower rigidity did not increase the stress at adjacent facet joints as much as the models with titanium rods (FUS model). When the fusion models were placed in extension and bending, the facet contact forces increased by 27–133% at L3–4 and increased by 4–26% at L2–3. The greatest increase in stress at the adjacent facet joints occurred in the FUS model when placed under a bending condition, increasing by 233% over the intact spine (INT). In contrast, using PEEK and biodegradable rods resulted in less of an increase in contact force at the adjacent levels. The situation mentioned above over time may result in facet hypertrophy and accelerate joint degeneration. Previous literature [10, 33, 34] indicated that a high rigidity at the instrumented level may lead to a number of biomechanical changes in the spine, such as elevated adjacent disc pressure, increased loading at facet joints, and increased mobility of adjacent segments. Altering the biomechanical environment of the spine in such a way may increase the risk of adjacent-level disease. This study also found that although the CageFUS model did show an increase in facet stresses at adjacent levels, the increase was less than all other models with instrumentation. This demonstrates that removing the pedicle screws might reduce the incidence of adjacent segment disease.

When applying posterior instrumentation during interbody fusion, stress-shielding limits the loads transferred to bone grafts at the interbody space. Rods with lower rigidity may offer less stress-shielding between two vertebral bodies, meaning that the fusion site might receive greater contact stress. The greater contact stress may be beneficial to the fusion process in accordance with Wolff’s law. An animal study by Dijk et al. [35] suggested that lowering the level of stress shielding could increase the rate of fusion. This current study demonstrated significantly higher intracage loading in the groups with semi-rigid fixation and without instrumentation, which implies lower stress-shielding at the fusion site. Therefore, using semi-rigid spinal fixators might not only mitigate adjacent diseases caused by the posterior instrumentation, but also promote fusion at the index level.

There are some limitations to this study that should be declared. First, a specific single-level interbody fusion (L4–5) was simulated, but the fusion conditions at other levels of the lumbar spine were not analyzed. Second, the shape of the vertebral bodies was simplified to be similar in shape, but the size of each body and disc was scaled according to x-ray images. The vertebrae were also assumed as homogenous and isotropic structures, which is not a truly accurate simulation of the anatomical vertebrae. Third, the loading conditions were not representative of truly physiological loading conditions, because these models could not simulate the mechanical effect of muscle contraction. Also, with the use of the hybrid method [22], the moment placed on the fusion segment increases proportionally to the additional adjacent segment motion. Therefore, adjacent segments must compensate more when using rigid implants than mobile devices [36]. Fourth, the stiffness of the adjacent segments directly impacts the motion distribution among these segments. Due to its nonlinear behavior, the spine offers low resistance to movement when in its neutral position, but gradually stiffens when loaded. This means that the stiff adjacent segments will typically have a lower range of motion than mobile segments. Therefore, even though all segments are subjected to the same loading, the mobility of adjacent segments may vary [36]. The human spine is a structure with complex geometry and a variety of material properties and boundary conditions, and so the finite element method is suitable for evaluating the biomechanical effect on the facet joints and discs at the index and adjacent levels after instrumentation and implantation of cages. Moreover, the finite element method often provides advantages when individual variations exist because it allows cause-effect relationships to be isolated and fully explored. Fifth, the failure of fusion was not considered in this study, a revision surgery should be performed if the rod absorbed prior to fusion being complete and the vertebral was determined as unstable.

## Conclusion

The findings of our study suggest that the ROM and adjacent disc stress were not significantly affected by using different rod materials as spinal fixators for interbody fusion with a PEEK cage. Using flexible rods or just using a cage alone could reduce the relative increase in contact force at adjacent facet joints and provide less stress shielding between two instrumented bodies. The removal of the posterior spinal fixator after the fusion process was finished could be beneficial for reducing loading on adjacent facet joints and alleviating hardware related discomfort. Due to the insufficient stability under rotational motions, we do not recommend removing the posterior spinal fixator too early until complete fusion has occurred.

## Data Availability

The datasets used and/or analyzed during the current study are available from the corresponding author upon reasonable request.

## Abbreviations

ASDs: Adjacent segment diseases
BIOFUS: Spine implanted with a lumbar cage and pedicle screws with a biodegradable rod system at L4-L5
CageFUS: Spine implanted with a lumbar intervertebral cage at L4-L5 without pedicle screws or rods (interbody fusion without pedicle screw system)
FUS: Spine implanted with a lumbar cage and pedicle screws with a Ti-6Al-4 V rod system at L4-L5
INT: Intact spine
IVDs: Intervertebral discs
PEEKFUS: Spine implanted with a lumbar cage and pedicle screws with a PEEK rod system at L4-L5
ROM: Range of motion
